# Rheumatic fever in a developed country – is it still relevant? A retrospective, 25 years follow-up

**DOI:** 10.1186/s12969-022-00678-7

**Published:** 2022-03-15

**Authors:** Rotem Tal, Mohamad Hamad Saied, Razi Zidani, Yoel Levinsky, Rachel Straussberg, Jacob Amir, Gil Amarilyo, Liora Harel

**Affiliations:** 1 Pediatric Rheumatology Unit, Schneider Children’s Medical Center of Israel, 4920235 Petach Tikva, Israel; 2 Sackler Faculty of Medicine, Tel Aviv University, Tel Aviv, Israel; 3 Pediatric Neurology Institute, Schneider Children’s Medical Center of Israel, Petach Tikva, Israel; 4 Department of Pediatrics, Maynei Hayeshua Medical Center, Bnei Brak, Israel

**Keywords:** Rheumatic fever, Pediatric rheumatology, Rheumatic heart disease

## Abstract

**Background:**

Our aims were to clinically and epidemiologically characterize rheumatic fever (RF) in the current era in Israel. Although there has been a steady decline in the incidence of RF in the western world, evidence of disease resurgence in developed countries continues to be published. The paucity of recent epidemiological data prompted our study.

**Methods:**

Medical files were retrospectively reviewed for all children with RF in our tertiary pediatric university-affiliated hospital from 1993 to 2017. Main outcome measures were patients and disease related characteristics, incidence trends, risk factors, disease course, relapse rates and secondary prophylaxis.

**Results:**

The cohort included 307 children. Sixty-four percent presented with arthritis, interestingly including hips and small joints of hands and feet at presentation, 52% presented with carditis. Severe carditis developed in 31 patients (19.5%), of whom 21 (13.2% of all carditis patients) acquired heart failure, 5 required intensive care monitoring, with one recent death. The percentage of patients with acute carditis of the overall RF patients remained relatively stable. Thirty-two patients (10% of patients with RF) relapsed, including 11 with a cardiac relapse (3.6% of all cardiac patients). The recurrence rate of RF continued to rise up to 9 years from the initial episode. One of 147 patients (< 0.7%) with a non-cardiac initial presentation had carditis at relapse.

**Conclusion:**

RF and rheumatic heart disease remain an important cause of morbidity and mortality including developed countries, with relapse rate continuing after 9 years of prophylaxis. Presentation of small joints as well as hips, although uncommon, should not exclude the diagnosis.

## Introduction

Rheumatic fever (RF) is an inflammatory process triggered by group A beta hemolytic streptococcal (GAS) tonsillopharyngitis [1]. Diagnosis depends on the presence of two major Jones criteria or one major and two minor criteria, with the abovementioned GAS infection being a prerequisite to diagnosis [2]. The cardiac system is not the most often involved, but it is the most important in terms of morbidity and mortality.

RF and rheumatic heart disease (RHD) pose a global health burden, with an estimated 470,000 new cases of acute RF per year, of which 60% lead to rheumatic heart disease [3]. The last few decades show a continuous decline in the incidence of RF in the western world, attributable to rapid treatment of GAS infections as well as general improvements in socioeconomic status [4]. Nevertheless, evidence of disease resurgence in developed countries continues to be published, including in the intermountain area of the Unites States [5], Europe [6, 7], and Australia [8, 9].

In 2015, the American Heart Association published the revised Jones criteria. The changes stratified populations into low, medium and high risk for acute RF and included echocardiographic definitions of carditis. The revised guidelines set more lenient criteria for the medium- high risk populations in an effort to decrease RF underestimations [2]. A retrospective study of 93 Indian patients found that the use of the modified 2015 Jones criteria increased the rate of diagnosis of RF from 66 to 86% [10].

Acute RF continues to be the most common cause of acquired heart disease in the developing world [11].

The paucity of recent epidemiologic data on acute RF in developed countries prompted the present study in Israel, where acute RF remains endemic. The aim of the study was to document patient- and disease-related parameters for all patients diagnosed with RF over the last 25 years at a major tertiary pediatric university-affiliated medical center in central Israel and to calculate the incidence and relapse rates.

## Methods

### Patients and setting

A retrospective cohort design was used. Data were collected from the medical records of all patients up to 18 years of age diagnosed with acute RF at the Schneider Children’s Medical Center of Israel located in the central urban area of the country from 1993 to 2017. All patients underwent assessment by a cardiologist, including echocardiography. The charts were reviewed to ensure adherence to the revised 2015 Jones criteria [2] with particular emphasis on monoarthritis to ensure the correct diagnosis. Patients who did not meet the criteria were excluded, as were patients with co-morbidities, including background rheumatological, neoplastic and cardiac diagnoses. Treatment and concomitant prophylaxis was in accordance with the American Heart Association recommendations. Elevated levels of Antistreptolysin O (ASLO) were defined according to our local laboratory references levels.

The study was approved by the local Helsinki Committee (REB no. 0253-17-RMC).

### Statistical analysis

Categorical variables are described as frequency (%), and ordinal variables, as median and interquartile range. Continuous variables were tested for normal distribution using histograms and Q-Q plots. Normally distributed continuous variables are described as mean and standard deviation, and non-normally distributed variables, as median and interquartile range. The rate of disease recurrence over time was determined using Kaplan-Meier curves. Median follow-up time was calculated using the reverse sensory method.

Univariate Cox regression was used to test the association between each predictor and disease recurrence in general and with cardiac recurrence in particular. One-sample t-test was used to compare the number of members per household in the cohort to the average number in the general population throughout the study period according to the published data of the Israel Central Bureau of Statistics [12, 13]. Chi-squared test was used to determine the association between antibiotic treatment during pharyngitis, gender and carditis. Patients with or without carditis were compared by age using independent-samples t-test, and by inflammatory markers, using Mann-Whitney U test. Spearman’s rank correlation coefficient was used to describe trends in the presentation of patients with RF, the presentation of patients with carditis, and the incidence of carditis among all patients with RF.

All statistical analyses were performed with the IBM SPSS statistical package (SPSS 24 Inc., Chicago, IL, USA).

## Results

### Demographic data

Of the 402 patients whose records were screened, 307 were included in the study. Basic demographic and clinical characteristics of the patients are depicted in Table 1. There were 188 male (61%) and 119 female patients (39%) of mean age 9.73 ± 3.45 years (range 2.3-17.5 years) at diagnosis. A family history of acute RF or RHD was documented in 70 patients (23%). Patients represented 85 geographical areas with a varied ethnic and socio-economic background including 57% Jews of European, Mediterranean and Ethiopian origin, as well as Arab children from various cities. Our study population resided in households with a median of 6 family members (range 5-8 members), compared to 3.4 in the general population for the same time period. A larger household was significantly associated with an increased incidence rate of acute RF (*P* < 0.001).

**Table 1:** Basic demographic and clinical characteristics of the patients with acute rheumatic fever

Parameter	At presentation	At relapse	*P*-value
Total number	307	32	NA
Females	119 (39%)	14 (44%)	0.575
Mean age at onset (years) ± SD (range)	9.73 ± 3.45 (2.3-17.5)	10.9 ± 3.6 (2.9-16.9)	0.0700
A family history of acute rheumatic fever	70 (23%)	6 (19%)	0.824
Duration of hospitalization (days, mean)	8.83 ± 6.91	10.37 ± 6.9	0.2570
Ethnicity – Jewish	177 (53%)	11 (52%)	0.7339
Arab	34 (11%)	3 (14%)	
Duration of follow-up (months), median ± SD (range)	49 ± (13-102)	32 (22-87)	NA
**Streptococcal exposure**			
Recent pharyngitis	175 (57%)	10 (38%)	0.099
Positive throat culture	143 (47%)	20 (63%)	0.548
Elevated ASLO titer	238 (78%)	22 (71%)	0.3717
Median time from onset of pharyngitis to hospital admission (days, mean)	22.32 ± 19.8	25.6 ± 20.4	0.3747
**Clinical manifestations – major criteria**			
Arthritis	197 (64%)	20 (63%)	0.849
Carditis	160 (52%)	11 (34%)	0.064
Chorea	47 (15%)	5 (16%)	0.900
Erythema marginatum	16 (5.2%)	1(3.1%)	1.000
Subcutaneous nodules	2 (0.7%)	1 (3.1)	0.258
**Minor criteria**			
Fever	94 (32%)	17 (53%)	0.016
Arthralgia	229 (75%)	11 (34%)	> 0.001
Prolonged PR	23 (7.5%)	3 (9.4%)	0.724
Elevated CRP	269 (87%)	24 (75%)	0.058
CRP (mean ± SD)	8.6 ± 7.7	11.2 ± 8.6	0.0952
Elevated ESR	278 (91%).	27 (84%)	0.347
ESR (mean ± SD)	79.7 ± 28	81.6 ± 31	0.7453

*NA* Not applicable

Arthritis was the most common sign, manifesting in 197 patients (64%).Patients had a mean of 3 ± 1.75 involved joints (range 1-10), particularly the knees (63%) and ankles (57%). The arthritis was migratory in 152 patients (77%). Twenty patients (10%) had monoarthritis. Involvement of the small joints of the hands and feet was noted in 28%, and initial hip involvement in 20%. Patient with hip involvement were less likely to have carditis compared to patients with arthritis of other joints [11(27.5%) versus 67 (45%), respectively, *P* = 0.049].

Table 2 describes the rate of joint involvement.

**Table 2:** Characteristics of joint involvement among the cohort

Parameter	At presentation	At relapse	*P*-value
Migratory arthritis	152 (77%)	16 (80%)	0.461
Mono arthritis	21 (10%)	1 (5.0%)	0.701
**Distribution**			
Knee	124 (63%)	12 (60%)	0.812
Ankle	113 (57%)	8 (40%)	0.160
Small joints of hands and feet	55 (28%)	7 (35%)	0.604
Hip	41 (21%)	6 (30%)	0.392
Wrist	30 (15%)	2 (10%)	0.745
Elbow	24 (12%)	4 (20%)	0.301
Spine	14 (7.1%)	1 (5.0%)	1.000
Shoulder	12 (6.1%)	2 (10%)	0.624

Carditis was found in 160 patients (52%); 147 (92%) had mitral valve insufficiency and 109 (68%), aortic valve insufficiency. Other valve manifestations included tricuspid regurgitation (2%), mitral stenosis (2%), pulmonary valve incompetence (0.6%), and moderate aortic stenosis (0.3%). Steroid therapy was given to 31 patients with severe carditis (19.5% of carditis patients; 10% of all patients). The remaining carditis patients received acetylsalicylic acid. Cardiac failure developed in 21 patients (13% of patients with cardiac involvement) Five of these patients required admission to the intensive care unit (during the years 2017-2018); one patient died (0.3% overall mortality), a fifteen-year-old male with trisomy 21 and normal previous cardiac screen. He presented with fever, elevated inflammatory markers and severe carditis, with consequent rupture of chordae tendinae of the mitral valve. He passed away during urgent surgical valve replacement attempt. Patients who had carditis were significantly older than those who did not (mean age 10.28 ± 3.14 years vs 8.97 ± 3.66 years, *P* < 0.001). Residual heart disease was found in 64% of the patients with carditis.

Inflammatory markers were markedly elevated. At admission, CRP was positive in 269 patients (87.6%) and erythrocyte sedimentation rate (ESR) was elevated in 278 (90.5%).

Chorea was found in 47 patients (15%). Chorea was present for a mean of 218.5 ± 482.8 days. It was twice as common in girls (*P* = 0.01).

Skin manifestations were rare: 16 patients (5.2%) had erythema marginatum and 2 (0.7%) had subcutaneous nodules.

### Antecedent GAS infection and antibiotic treatment

Recent pharyngitis was reported by 175 patients (57%), of whom, according to patient reports, 63 (36%) received appropriate antibiotic therapy, 44 (25%) received partial treatment, and the remainder were untreated. The median time from onset of pharyngitis to hospital admission was 14 days (mean 22.32 ± 19.8 days).

### Hospitalization and recurrence

The mean duration of hospitalization was 8.83 ± 6.91 days, and the average follow-up time per patient was 49 months (range 13-102 months). The rate of recurrence of RF varied by duration of follow-up. Of the 268 children who were followed continuously in our hospital, 32 had a recurrence (18 patients receiving oral therapy, 13 patients receiving parenteral therapy). After taking into account patients lost to follow up, recurrence rate was 8.7% after 2 years, 13.9% at 5 years, and 21.2% after 9 years. Details regarding clinical features at relapse are presented in Tables 1 and 2.

Carditis recurred in 11 of the 268 patients (4%) under long-term follow-up. In 10 of them, carditis was part of the initial presentation. The remaining patient, in whom carditis was part of relapsed RF, had only non-cardiac symptoms at presentation: fever, migratory arthritis, elevated inflammatory markers and positive anti-streptococcal serology. His initial echocardiogram was normal, and he was discharged after 9 days with oral antibiotics. He returned after 11 months with a similar presentation, this time including moderate, asymptomatic, mitral valve incompetence. At discharge, he was referred for parenteral antibiotic prophylaxis. His most recent cardiac assessment performed 26 months later, showed minimal residual mitral valve incompetence.

**Fig. 1:**
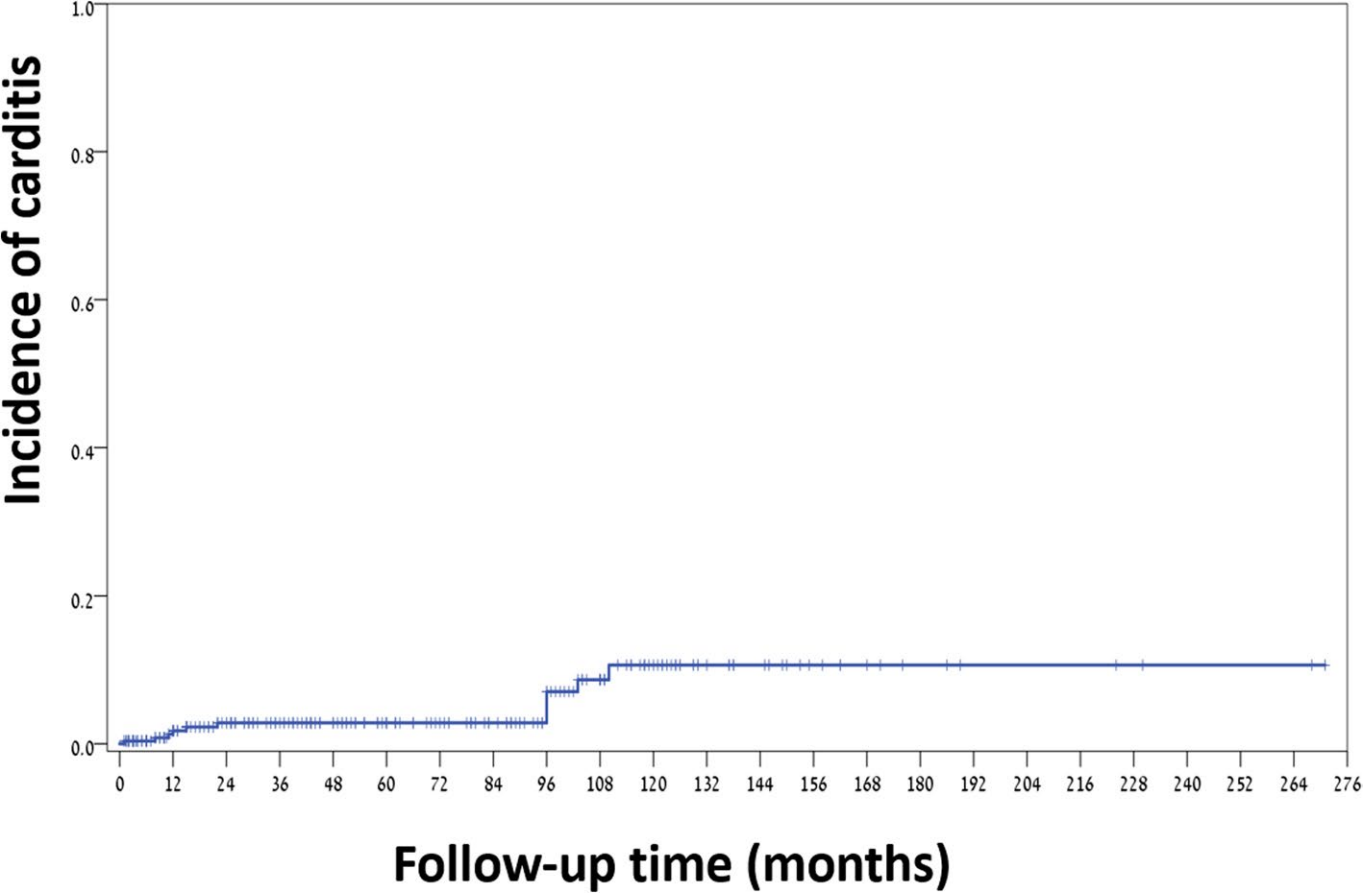
Recurrence rates of carditis in patients previously diagnosed with Rheumatic Fever in our study

The median age of patients at the time of carditis recurrence was 9.8 years (range 7-12). Older age at disease onset was associated with a lower risk of cardiac recurrence [HR older age at onset = 0.79, 94%CI 0.63-0.99, *P* = 0.043). Figure 1 shows the recurrence rate of RF with carditis.

### Incidence of RF and carditis over time

**Fig. 2:**
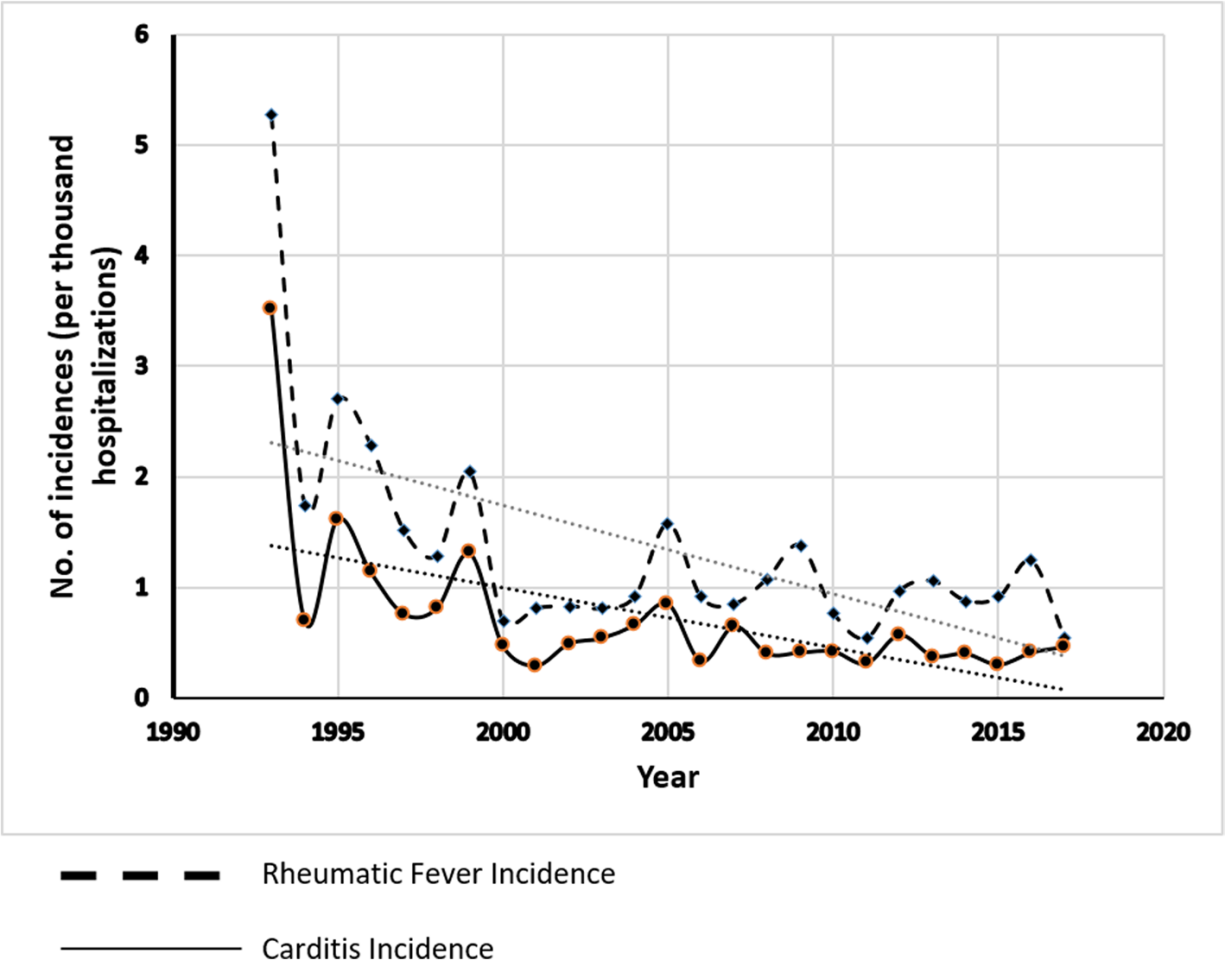
Rheumatic fever and carditis incidence proportion of overall hospitalized patients

**Fig. 3:**
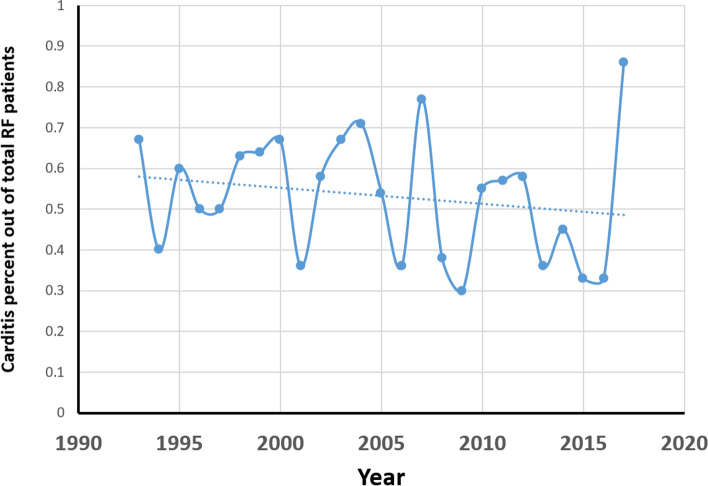
Proportion of Carditis Patients Out of All Rheumatic Fever Cases

The incidence rate of RF was calculated relative to the overall admissions in our hospital during the study period, with a final incidence per 1000 general admissions. Over the 25 year period of the study, the incidence of RF at our center ranged from 5.27 per 1000 in 1993 to 0.53 per 1000 in 2017. There was a general decrease in the incidence of both rheumatic fever (*r* = − 0.544, *P* = 0.005) as well as carditis (*r* = 0.698, *P* < 0.001), demonstrated in Fig. 2. However, there was no decrease in the incidence of carditis as the initial presentation of RF during the study period (*r* = − 0.274, *P* = 0.184), Fig. 3.

## Discussion

This is the most expansive hospital study of Acute RF in Israel, spanning 25 years in a pediatric tertiary care center.

### Rheumatic fever incidence

Globally, RF remains a significant contributor to morbidity, particularly in developing countries. The annual incidence varies widely from 0.5 per 100,000 in the USA, escalating to 8 per 100,000 to 51 per 100,000 in children and young adults in developing countries [14–16]. The global age-standardized mortality rate of RHD decreased by 47.8% from 1990 to 2015. In 2015, there were 33.4 million cases of RHD and 10.5 million disability-adjusted life years secondary to RHD globally [16].

The most recent study by Vinker et al [17] in 2010 reported an annual incidence of rheumatic fever in Israel of 7.5 per 100,000 population. The current study at our tertiary referral center in central Israel covers the period from 1993 to 2017. The incidence has declined from 5.27 to 0.54 per 1000 admissions, likely reflecting the general decrease in incidence illustrated in developed countries.

The worldwide decline in RF prevalence during the last decades is multifactorial. These include improvement in factors that influence the transmission of GAS such as access to high-quality and prompt health care, education and household overcrowding [4, 16, 18]. Jaine et al. reported that in New-Zealand, RF rates were associated with household crowding and that this effect persisted after controlling for the density of children in the neighborhood [18]. Israel is one of the most densely populated countries in the world, and its level of population density continues to escalate. It is estimated to become the most densely populated country in the world by 2040 [19]. Nevertheless, its household crowding gradually decreased through the years 1980-2019 [20]. This data support the findings from New-Zealand and strengthen the importance of household crowding as a major risk factor for GAS transmission and RF incidence.

### Epidemiologic factors

The male: female ratio of affected patients was 2:3 as reported by the World Health Organisation [21] in contrast to a recent report of 1:1 ratio among the USA population [22]. Our patient population spanned a wide geographical and ethnic range. The significantly high number of members per household compared to the average Israeli household confirmed relative overcrowding as an independent risk factor for RF [23]. According to the recent USA report by Loizaga et al., children living in more deprived communities are at increased risk of severe RHD. This may reflect living conditions such as overcrowding, poor sanitation, and poor hygiene, which may serve as risk factors to exposure to group A streptococcus [22].

Twenty-three percent of our patients had a family history of RF /RHD. We assume this to be at least partially influenced by cultural and socio-economic similarities. There is a known genetic susceptibility conferred by polymorphism of genes involved in innate and adaptive immune pathways [24]. Our patients did not undergo genetic assessment.

The long duration of hospitalization (mean 8.83 ± 6.91 days) emphasizes the previously reported, substantial health and financial strain commencing in the acute phase of this disease.

More than half the patients (57%) had a history of pharyngitis, of which 64% received inadequate or no antibiotic therapy, highlighting the importance of adherence to primary prophylaxis regimen.

### Arthritis

Regarding arthritis, interestingly 20% presented with initial hip involvement, a relatively less commonly involved joint [25]. These patients had statistically significant less carditis as compared to other joint involvement. Arthritis was non-migratory in 15%. 11% of patients had monoarthritis. No anti-inflammatory medications were administered that may otherwise explain this joint manifestation as an aborted attack. Small joints of the hands and feet were involved at initial presentation in 29% of patients. In contrast to hip involvement, there was no difference between cardiac involvements when comparing them to typical joint presentations.

### Carditis

In our study, the incidence of carditis decreased throughout the study period in parallel to the general decrease in the incidence of RF. However, the proportion of carditis cases among patients with RF remained unchanged. All patients were recommended continued penicillin prophylaxis.

In view of the global burden of rheumatic heart disease, our findings emphasize the importance of bearing a RF diagnosis in mind and screening for cardiac involvement despite the decreasing global incidence, particularly in developed countries.

### RF recurrence

RF recurred in 32 patients (8.7% within the first 2 years and 13.9% by 5 years). The rate of recurrence increased steadily to 21.2% at 9 years, highlighting the importance of continued long-term prophylaxis. No further increase in rate was shown after 9 years. The rate of recurrence was 15% in patients on oral antibiotic prophylaxis compared to 9% in patients on parenteral prophylaxis (HR =0.524 for parenteral prophylaxis). This finding supports previous data showing improved compliance and less recurrence in patients on parenteral vs oral therapy [26]. Most of the patients with recurrent carditis had evidence of re-infection as per ASLO or positive throat culture. Due to the retrospective nature of our study, we have no direct way of knowing their compliance or lack of, but compliance may have been lower in these patients. It should be noted that although adherence to prophylaxis is unknown, we previously showed overall poor adherence to secondary prophylaxis to penicillin in Israel [27].

### Limitations

Our study was limited by the biases inherent to a retrospective study. The setting of the study in a tertiary medical center posed a risk of selection bias. We were also unable to confirm antibiotic compliance, particularly in the oral prophylaxis group.

## Conclusions

To our knowledge, this is the largest study of pediatric RF in a developed country to date, spanning 25 years. Presentation of small joints as well as hips should not exclude the diagnosis. Initial hip arthritis was associated with less cardiac involvement. Only one patient with carditis at relapse did not have carditis at initial presentation. The findings emphasize that ARF and RHD remain an important cause of morbidity and mortality, with relapses continuing after 9 years, highlighting the need for prolonged prophylaxis.

## Data Availability

The datasets used and/or analysed during the current study are available from the corresponding author on reasonable request.

## Abbreviations

RF: Rheumatic fever (RF)
GAS: Group A beta hemolytic streptococcus
RHD: Rheumatic heart disease
ASLO: Antistreptolysin O

## References

[1] Carapetis JR, McDonald M, Wilson NJ (2005). Acute rheumatic fever. Lancet.

[2] Gewitz MH, Baltimore RS, Tani LY (2015). American Heart Association Committee on rheumatic fever, endocarditis, and Kawasaki disease of the council on cardiovascular disease in the young. Revision of the Jones criteria for the diagnosis of acute rheumatic fever in the era of Doppler echocardiography: a scientific statement from the American Heart Association. Circulation.

[3] Carapetis JR, Steer AC, Mulholland EK (2005). The global burden of group a streptococcal diseases. Lancet Infect Dis.

[4] Carapetis JR (2007). Rheumatic heart disease in developing countries. N Engl J Med.

[5] Veasy LG, Tani LY, Hill HR (1994). Persistence of acute rheumatic fever in the intermountain area of the United States. J Pediatr.

[6] Pastore S, De Cunto A, Benettoni A (2011). The resurgence of rheumatic fever in a developed country area: the role of echocardiography. Rheumatology.

[7] Munteanu V, Petaccia A, Contecaru N (2018). Pediatric acute rheumatic fever in developed countries: neglected or negligible disease? Results from an observational study in Lombardy (Italy). AIMS Public Health.

[8] Lawrence JG, Carapetis JR, Griffiths K (2013). Acute rheumatic fever and rheumatic heart disease: incidence and progression in the Northern Territory of Australia, 1997 to 2010. Circulation.

[9] Australian Institute of Health and Welfare. Acute rheumatic fever and rheumatic heart disease in Australia 2019; v5.0 [updated 2019 Oct 11]. Available from: https://www.aihw.gov.au/reports/indigenous-australians/acute-rheumatic-fever-rheumatic-heart-disease/contents/summary.

[10] Kumar D, Bhutia E, Kumar P (2016). Evaluation of the American Heart Association 2015 revised Jones criteria versus existing guidelines. Heart Asia.

[11] Dass C, Kanmanthareddy A. Rheumatic Heart Disease. 2021 Jul 31. In: StatPearls Treasure Island (FL): StatPearls Publishing; 2022. 30855870

[12] Israel Central Bureau of Statistics. http://www.cbs.gov.il/www/publications15/meshek_bait/mb_years.pdf. Accessed 26 Feb 2022.

[13] Israel Central Bureau of Statistics. http://www.cbs.gov.il/publications/social_survey03a/pdf/prt02.pdf . Accessed 26 Feb 2022.

[14] Marijon E, Mirabel M, Celermajer DS, Jouven X (2012). Rheumatic heart disease. Lancet.

[15] Karthikeyan G, Guilhereme L (2018). Acute rheumatic fever. Lancet.

[16] Watkins DA, Johnson CO, Colquhoun SM (2017). Global, regional, and national burden of rheumatic heart disease, 1995-2015. N Engl J Med.

[17] Vinker S, Zohar E, Hoffman R (2010). Incidence and clinical manifestations of rheumatic fever: a 6 year community-based survey. Isr Med Assoc J.

[18] Jaine R, Baker M, Venugopal K (2011). Acute rheumatic fever associated with household crowding in a developed country. Pediatr Infect Dis J..

[19] Israel Central bureau of statistics, Projections of Israel Population until 2065. https://www.cbs.gov.il/he/mediarelease/DocLib/2017/138/01_17_138b.pdf. Accessed 26 Feb 2022.

[20] Israel Central bureau of statistics, Households – Economic Characteristics and Housing Density. https://www.cbs.gov.il/he/publications/doclib/2021/households_eco19_1816/t_01.pdf. Accessed 26 Feb 2022.

[21] World Health Organization. Seventy First World Health Assembly Provisional agenda item 12.8, A71/25: 12 April 2018. Rheumatic Fever and rheumatic heart disease. Report by the Director-General. Available from: https://apps.who.int/gb/ebwha/pdf_files/WHA71/A71_25-en.pdf.

[22] de Loizaga SR, Arthur L, Arya B, Beckman B, Belay W, Brokamp C (2021). Rheumatic heart disease in the United States: forgotten but not gone: results of a 10 year multicenter review. J Am Heart Assoc.

[23] Nascimento BR, Beaton AZ (2019). Rheumatic heart disease and socioeconomic development. Lancet Glob Health.

[24] Muhamed B, Parks T, Sliwa K (2020). Genetics of rheumatic fever and rheumatic heart disease. Nat Rev Cardiol.

[25] Pileggi GC, Ferriani VP (2000). Atypical arthritis in children with rheumatic fever. J Pediatr.

[26] Manyemba J, Mayosi BM (2003). Intramuscular penicillin is more effective than oral penicillin in secondary prevention of rheumatic fever--a systematic review. S Afr Med J.

[27] Amarilyo G, Chodick G, Zalcman J (2019). Poor long-term adherence to secondary penicillin prophylaxis in children with rheumatic fever. Semin Arthritis Rheum.

